# The Polyproline Site in Hinge 2 Influences the Functional Capacity of Truncated Dystrophins

**DOI:** 10.1371/journal.pgen.1000958

**Published:** 2010-05-20

**Authors:** Glen B. Banks, Luke M. Judge, James M. Allen, Jeffrey S. Chamberlain

**Affiliations:** 1 Department of Neurology, Senator Paul D. Wellstone Muscular Dystrophy Cooperative Research Center, University of Washington, Seattle, Washington, United States of America; 2 Department of Medicine, University of Washington, Seattle, Washington, United States of America; 3 Department of Biochemistry, University of Washington, Seattle, Washington, United States of America; The Jackson Laboratory, United States of America

## Abstract

Mutations in dystrophin can lead to Duchenne muscular dystrophy or the more mild form of the disease, Becker muscular dystrophy. The hinge 3 region in the rod domain of dystrophin is particularly prone to deletion mutations. In-frame deletions of hinge 3 are predicted to lead to BMD, however the severity of disease can vary considerably. Here we performed extensive structure-function analyses of truncated dystrophins with modified hinges and spectrin-like repeats in *mdx* mice. We found that the polyproline site in hinge 2 profoundly influences the functional capacity of a microdystrophin^ΔR4-R23/ΔCT^ with a large deletion in the hinge 3 region. Inclusion of polyproline in microdystrophin^ΔR4-R23/ΔCT^ led to small myofibers (12% smaller than wild-type), Achilles myotendinous disruption, ringed fibers, and aberrant neuromuscular junctions in the *mdx* gastrocnemius muscles. Replacing hinge 2 of microdystrophin^ΔR4-R23/ΔCT^ with hinge 3 significantly improved the functional capacity to prevent muscle degeneration, increase muscle fiber area, and maintain the junctions. We conclude that the rigid α-helical structure of the polyproline site significantly impairs the functional capacity of truncated dystrophins to maintain appropriate connections between the cytoskeleton and extracellular matrix.

## Author Summary

Dystrophin functions like a large molecular spring between the muscle cytoskeleton and the extracellular matrix in order to protect the membrane from contraction-induced injury. Mutations in dystrophin can lead to a severe muscle wasting disease called Duchenne muscular dystrophy (DMD) in young boys. DMD patients are typically wheelchair bound by 9–13 years of age and die at approximately 30 years. There are also mutations within the dystrophin gene that lead to internal truncations of non-essential regions, such as the internal rod domain that leads to a mild form of the disease called Becker Muscular Dystrophy. However, these internal truncations frequently occur at a “hot spot” within the rod domain where the resulting disease severity is difficult to predict. Here we found that consecutive proline residues, that function much like a molecular ruler, can dramatically influence the function of these internally truncated dystrophins within skeletal muscles. Using this information, we designed a dystrophin mini-gene that can accommodate the limited packaging size of recombinant adeno-associated virus. This virus can deliver the dystrophin mini-gene to most muscles throughout a dystrophic mouse to prevent muscle degeneration and partially restore muscle function.

## Introduction

Duchenne muscular dystrophy (DMD) is a lethal X-linked recessive disease caused by mutations in the 2.2 MB dystrophin gene [1]–[3]. In skeletal muscle, dystrophin provides a flexible connection between the cytoskeleton and the dystrophin-glycoprotein complex at the sarcolemma, myotendinous junction (MTJ) and neuromuscular junction (NMJ) [4]–[6]. Mutations that affect the mechanical integrity of this molecular scaffold render muscles more susceptible to contraction-induced injury leading to cycles of necrosis and regeneration [3].

**Figure 1:**
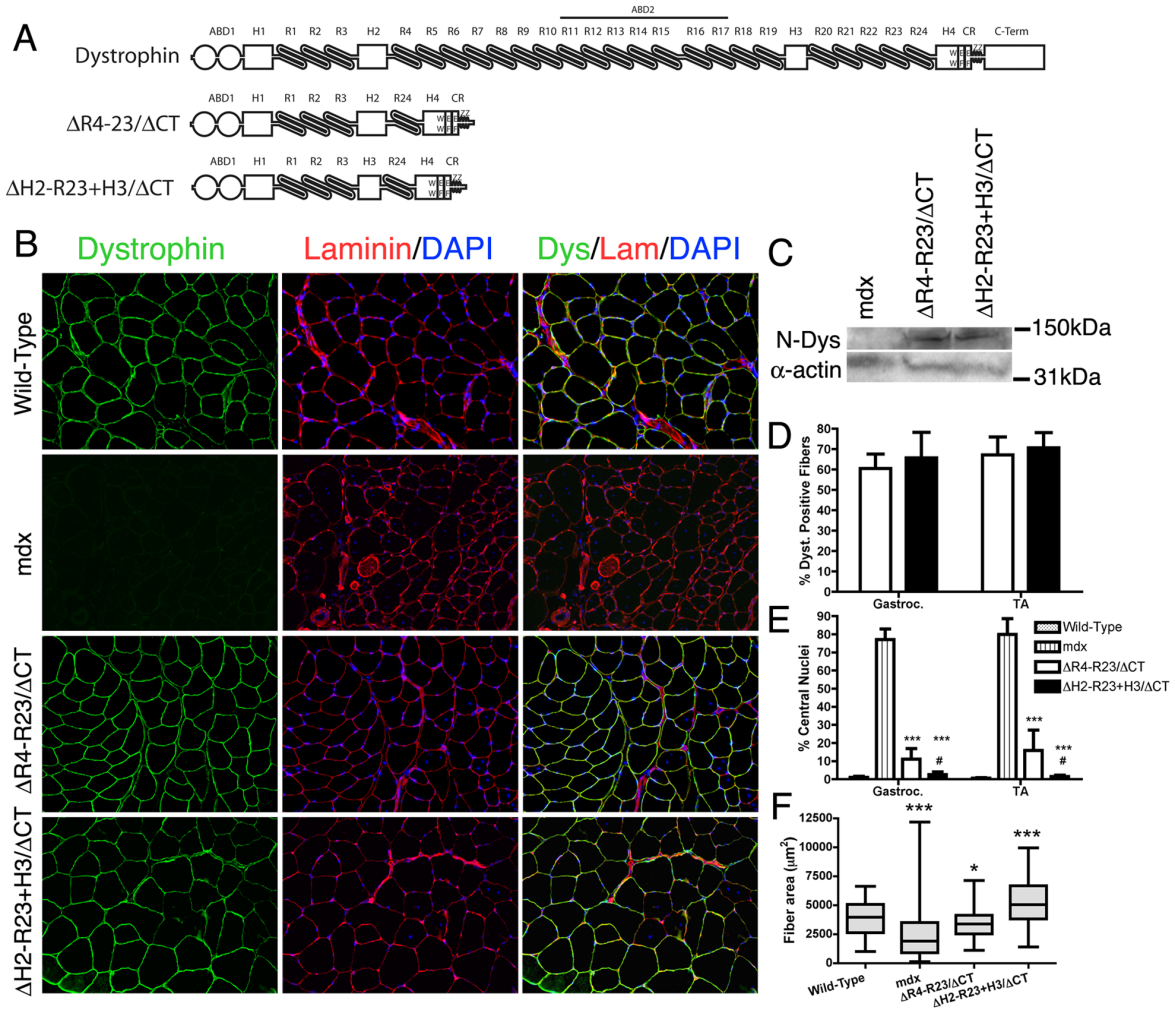
The hinge domains of dystrophin influence muscle maturation and maintenance. (A) The molecular structure of truncated dystrophins (reviewed in: [15]). ABD1 at the N-terminus is composed of two calponin homology domains denoted by the two circles. The central rod domain contains 24 spectrin-like repeats (R1-24), 4 hinge domains, a 20 amino acid insertion between spectrin-like repeats 15 and 16, and a central actin-binding domain (ABD2). A cluster of basic repeats forms ABD2 that bind to actin through an electrostatic interaction [20]. The hinge domains vary in that hinge 2 contains a polyproline site and hinge 4 contains a WW motif that is required for binding to β-dystroglycan [19], [68]. The cysteine-rich region contains two EF hands and a ZZ domain that is also required for binding to β-dystroglycan. The microdystrophins used in this study are shown below the full-length dystrophin. Microdystrophin^ΔR4-R23/ΔCT^ lacks a large portion of the central rod domain between spectrin-like repeats 4 and 23 and also lacks the C-terminal domain (ΔR4-R23/ΔCT) [24]. Note that microdystrophin^ΔR4-R23/ΔCT^ and microdystrophin^ΔH2-R23+H3/ΔCT^ differ by a single hinge domain. (B) Transverse sections of gastrocnemius muscles from wild-type, *mdx* and *mdx* mice expressing either microdystrophin^ΔR4-R23/ΔCT^ or microdystrophin^ΔH2-R23+H3/ΔCT^. Scale bar  = 100 µm. (C) Expression of truncated dystrophins in treated *mdx* gastrocnemius muscles was similar. The western blots were performed using frozen muscles extracted from OCT. All lanes were loaded equally as shown by α-sarcomeric actin. (D) Shown is the mean +/− S.D. percentage of dystrophin-positive muscle fibers. TA is tibialis anterior. (E) Shown is the mean +/− S.D. of the percentage of muscle fibers containing centrally-located nuclei. Microdystrophin^ΔH2-R23+H3/ΔCT^ with hinge 3 was more effective at preventing muscle degeneration in *mdx* mice compared to microdystrophin^ΔR4-R23/ΔCT^ with hinge 2. The treated muscles show the mean +/− SD for dystrophin-positive fibers only. ***P<0.001 compared to untreated *mdx* mice. ^#^ *P*<0.05 compared to microdystrophin^ΔR4-R23/ΔCT^ treated *mdx* muscles. (F) Hinge regions of dystrophin influence muscle fiber cross-sectional area. Shown is the mean +/− distribution (25th and 75th percentile (box) in addition to the farthest (whiskers) area of muscle fibers. *P<0.05 and ***P<0.001 compared to wild-type.

As a general rule, most frame-shift mutations in dystrophin lead to DMD whereas internal truncations (in-frame deletions) lead to a milder form of the disease called Becker muscular dystrophy (BMD) [7]–[14]. The severity of BMD can also vary depending on whether a critical region of dystrophin is deleted and the amount of dystrophin being expressed [7]–[14]. Dystrophin consists of a N-terminal actin-binding domain, a large central rod domain, a cysteine rich region and a C-terminal domain (Figure 1A) [15], [16]. The central rod domain contains 24 spectrin-like repeats, 4 hinges and a second actin-binding domain [15]–[20]. The locus encoding the N-terminal actin-binding domain and the region near hinge 3 of dystrophin are more susceptible to deletion mutations [7]–[13]. In-frame deletions of the central rod domain typically lead to a mild BMD [8]–[13]. However, in-frame deletions at the “hot spot” near hinge 3 can lead to more variable phenotypes [8]–[13], [21].

The role of dystrophin *in vivo* has been largely defined by the structure-function relationship of truncated dystrophins in humans and mice [8]–[13], [22]–[24]. Rational design of dystrophin mini-genes has been highly effective in preventing and reversing functional abnormalities of dystrophic muscles [22]–[29]. In particular, we previously developed a microdystrophin (ΔR4-R23/ΔCT; defined as those with 4 or fewer spectrin-like repeats [24]) that accommodates the limited cloning capacity of recombinant adeno-associated viral vectors (rAAV) [24]. Intravenous injection of rAAV vectors pseudotyped with serotype 6 capsid (rAAV6) expressing microdystrophin^ΔR4-R23/ΔCT^ can prevent and reverse most aspects of dystrophic pathology in *mdx* muscles [24], [28], [30]–[35]. Microdystrophin^ΔR4-R23/ΔCT^ also significantly protects muscles from contraction-induced injury [24], [28], [30]–[35].

While the microdystrophin^ΔR4-R23^ transgene provides a clear benefit to dystrophic muscles [24], more detailed analyses have revealed a potentially serious abnormality in some muscle groups. The microdystrophin^ΔR4-R23^/*mdx* transgenic mice have chronic Achilles myotendinous strain injury, which leads to the formation of ringed fibers and fragmentation of the neuromuscular junctions [33], [36]. In the present study we examined whether the domain composition or the small size of microdystrophin^ΔR4-R23/ΔCT^ led to this myopathy in *mdx* mice. We found that the hinge regions of microdystrophin, rather than its small size can profoundly influence skeletal muscle maintenance, maturation and structure.

## Results

### Dystrophin hinge domains influence the maintenance and maturation of skeletal muscles

We initially screened several truncated dystrophins and found that inclusion of hinge 2, but not hinge 3 could lead to the structural abnormalities we observed in some muscles of the microdystrophin^ΔR4-R23^ transgenic mice (Text S1; Figures S1, S2, S3). We subsequently compared the efficacy of two microdystrophins that differ only in their inclusion of hinge 2 (microdystrophin^ΔR4-R23/ΔCT^) or hinge 3 (microdystrophin^ΔH2-R23+H3/ΔCT^) (Figure 1A) to examine whether the hinge composition of microdystrophin could influence various aspects of muscle disease.

We administered a sub-optimal dose of 2×10^12^ vector genomes of a rAAV6 pseudotyped vector expressing either microdystrophin^ΔR4-R23/ΔCT^ or microdystrophin^ΔH2-R23+H3/ΔCT^ intravenously into 2 week-old *mdx^4cv^* mice. We used a sub-optimal dose of rAAV6-microdystrophins so that we could examine whether changing the hinge domain increased or decreased the functional capacity of microdystrophin. Six months after treatment, both microdystrophins were expressed in a similar percentage of gastrocnemius and tibialis anterior (TA) muscle fibers (ranging from approximately 61% to 71%; P = 0.238 when comparing between the microdystrophins; Figure 1B and 1D). Western blots confirmed similar expression levels of truncated dystrophins in treated gastrocnemius muscles (Figure 1C). Both microdystrophins restored dystrophin-associated proteins to the sarcolemma except for nNOS (Text S1; Figure S4). Microdystrophin^ΔR4-R23/ΔCT^ containing hinge 2 significantly prevented muscle degeneration (∼11% central nuclei for treated muscles verse ∼78% for untreated *mdx* muscles; *P*<0.001), and limited the fiber area of skeletal muscles (12% smaller than wild-type; *P*<0.05; Figure 1E), consistent with previous studies [24], [32], [33]. Microdystrophin^ΔH2-R23+H3/ΔCT^ containing hinge 3 was significantly better able to prevent muscle degeneration (1–2% central nuclei; P<0.05 compared to microdystrophin^ΔR4-R23/ΔCT^), and surprisingly increased average muscle fiber cross sectional area (34% larger than wild-type; P<0.001; Figure 1E). Thus, replacing hinge 2 of microdystrophin^ΔR4-R23/ΔCT^ with hinge 3 significantly improved its capacity to prevent muscle degeneration and promote skeletal muscle maturation.

### Dystrophin hinge domains influence myotendinous junction injury and formation of ringed fibers

**Figure 2:**
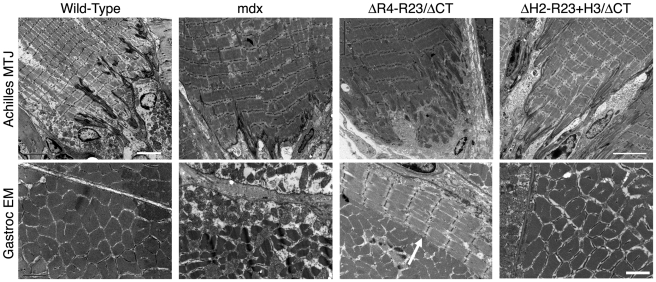
The hinge domains of dystrophin influence myotendinous junction structure and ringed fiber formation. Shown are electron microscopy images of longitudinal sections of the Achilles myotendinous junctions in addition to transverse sections of gastrocnemius muscles. Note that expression of microdystrophin disrupted the myotendinous junctions and led to formation of ringed fibers (arrow). Scale bar  = 5 µm for myotendinous junctions and 2 µm for transverse sections of gastrocnemius muscles.

The tendon extends deep folds into wild-type skeletal muscles to minimize membrane stress under shear (Figure 2)[37]. Most of the folds in the *mdx* junctions did not extend as far into the gastrocnemius muscles (Figure 2). rAAV6-microdystrophin^ΔR4-R23/ΔCT^ severely disrupted the Achilles myotendinous junctions in *mdx* mice. Many of the junctional folds were missing and myofibril degeneration was evident (Figure 2). Approximately 17% of the adjoining *mdx* gastrocnemius muscles had ringed fibers. In contrast, rAAV6- microdystrophin^ΔH2-R23+H3/ΔCT^ with hinge 3 retained the normal architecture of the Achilles myotendinous junction and we found no ringed fibers in the adjoining gastrocnemius muscles (Figure 2). Thus, the hinge domains influenced whether microdystrophin was capable of maintaining the myotendinous junction and myofibril structure in *mdx* gastrocnemius muscles.

### Dystrophin hinge domains influence neuromuscular synapse structure

**Figure 3:**
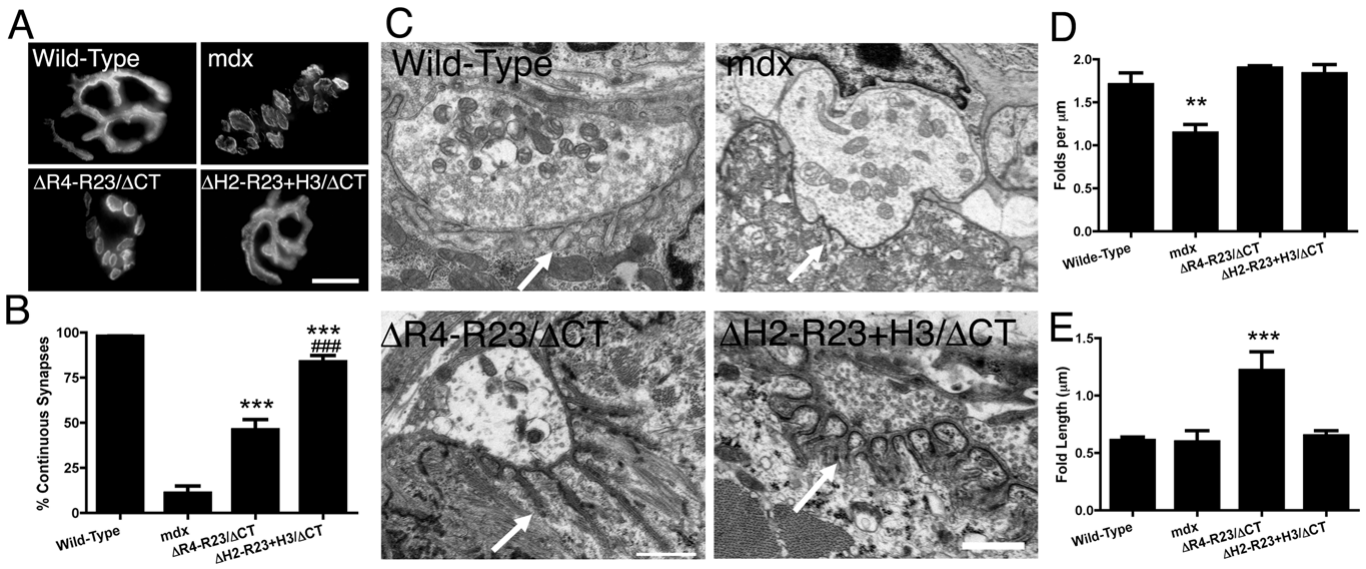
The hinge domains of dystrophin influence the structure of neuromuscular synapses. (A) Topographic view of AChR clusters stained with α-bungarotoxin. Scale bar  = 10 µm. (B) Graph shows mean +/− SD percentage of continuous synapses. Significant difference to *mdx* ***P<0.001. Significant difference to microdystrophin^ΔR4-R23/ΔCT^/*mdx* mice ^###^ *P*<0.001. (C) Ultrastructure of neuromuscular synapses from wild-type, *mdx* and *mdx* gastrocnemius muscles expressing microdystrophin^ΔR4-R23/ΔCT^ and microdystrophin^ΔH2-R23/ΔCT+H3^. Note that synaptic folding is reduced in *mdx* mice and increased in rAAV6-microdystrophin^ΔR4-R23/ΔCT^ treated muscles (arrows). Scale bar  = 0.5 µm. (D) The graph shows the mean +/− SD number of folds per µm of postsynaptic membrane juxtaposed to the presynaptic cleft. Significant difference compared to wild-type **P<0.01. (E) The mean +/− SD depth of the folds was significantly increased in *mdx* muscles treated with rAAV6-microdystrophin^ΔR4-R23/ΔCT^ (***P<0.001).

We also examined neuromuscular synapses in *mdx* mice treated with rAAV6-microdystrophins. Most neuromuscular synapses in wild-type mice (∼97%) form a continuous tertiary structure as shown by staining whole muscle fibers with α-bungarotoxin (Figure 3A). Neuromuscular synapses in *mdx* mice begin to fragment temporally coincident with muscle degeneration [38]. Approximately 89% of neuromuscular synapses were fragmented in the gastrocnemius muscles of *mdx* mice (Figure 3B). We had previously shown that the neuromuscular synapses in transgenic microdystrophin^ΔR4-R23^/*mdx* gastrocnemius muscles fragmented temporally coincident with the formation of ringed fibers [36]. In the present study we found that rAAV6- microdystrophin^ΔR4-R23/ΔCT^ containing hinge 2 maintained continuous synapses in only 46% of the *mdx* gastrocnemius muscles (Figure 3A and 3B). In contrast, approximately 84% of synapses were continuous in *mdx* gastrocnemius muscles treated with rAAV6-microdystrophin^ΔH2-R23+H3/ΔCT^ containing hinge 3 (Figure 3A and 3B).

Neuromuscular synapses also contain folds in the postsynaptic membrane that align directly adjacent to vesicle release sites (active zones) in the pre-synaptic nerve terminal (arrows; Figure 3C). The number of synaptic folds in *mdx* mice was significantly reduced compared to wild-type (P<0.01; Figure 3C and 3D) as previously described [4], [39]. The number of folds was restored in microdystrophin^ΔR4-R23/ΔCT^ and microdystrophin^ΔH2-R23+H3/ΔCT^ treated muscles (Figure 3C and 3D). The synaptic folds extended significantly further into microdystrophin^ΔR4-R23/ΔCT^ treated *mdx* muscles compared to wild-type muscles (*P*<0.001; Figure 3C and 3E), as previously described in transgenic microdystrophin^ΔR4-R23^/*mdx* mice [36]. In contrast, the number and length of synaptic folds in microdystrophin^ΔH2-R23+H3/ΔCT^ treated *mdx* muscles was similar to wild-type (Figure 3C–3E). Thus, microdystrophin^ΔH2-R23+H3/ΔCT^ containing hinge 3 can maintain the structure of neuromuscular junctions in *mdx* muscles.

### Mechanical properties of muscles expressing microdystrophins with either hinge 2 or hinge 3

**Figure 4:**
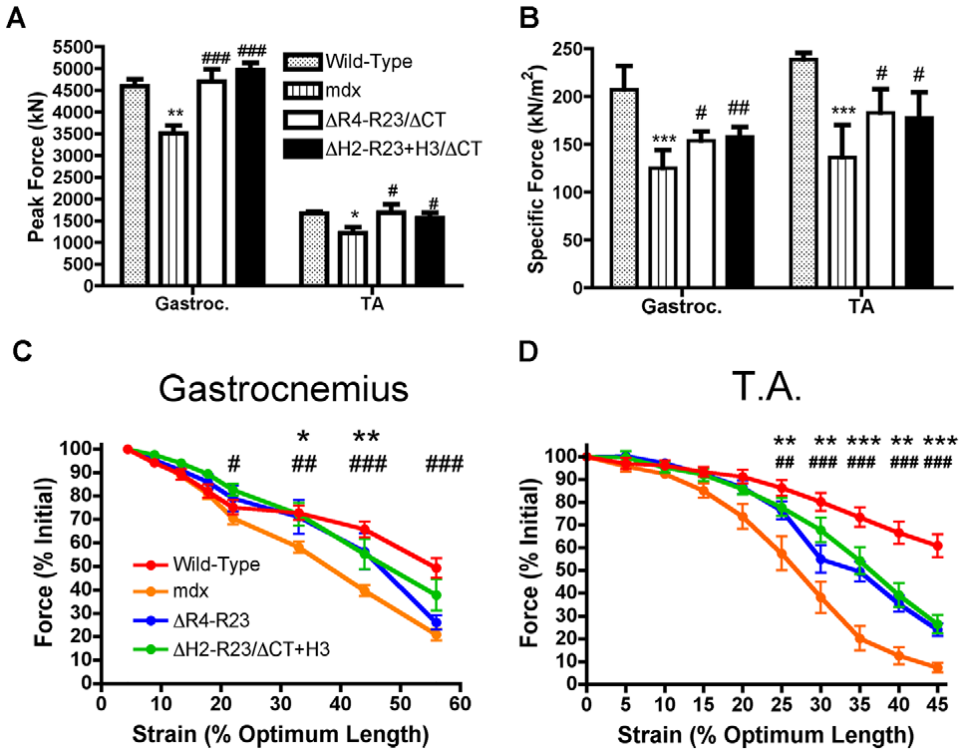
Microdystrophins significantly improve the mechanical properties of *mdx* hind limb muscles. (A) Graph shows the mean +/− S.D. peak force. *P<0.05 and **P<0.01 compared to wild-type. ^#^ *P*<0.05 and ^###^ *P*<0.001 compared to *mdx*. (B) Graph shows the mean +/− S.D. specific force. ***P<0.001 compared to wild-type. ^#^ *P*<0.05 and ^##^ *P*<0.01 compared to *mdx*. The contractile performance of (C) gastrocnemius muscles and (D) tibialis anterior muscles immediately prior to increasing length changes during maximal force production. Bars represent the mean +/− S.D. percentage of the initial optimal muscle contraction. rAAV6-microdystrophin^ΔR4-R23/ΔCT^ treated muscles were significantly (*P<0.05; **P<0.01; ***P<0.001) protected from contraction-induced injury, as were rAAV6-microdystrophin^ΔH2-R23/ΔCT+H3^ (^#^P<0.05; ^##^P<0.01; ^###^P<0.001) treated muscles when compared to *mdx* mice.

Contraction-induced injury can initiate muscle degeneration in *mdx* mice [40]. Skeletal muscles from *mdx* mice have a lower force producing capacity than wild-type muscles and are more susceptible to contraction-induced injury (Figure 4). We found that sub-optimal doses of both rAAV6-microdystrophin^ΔR4-R23/ΔCT^ and rAAV6- microdystrophin^ΔH2-R23+H3/ΔCT^ maintained the peak force producing capacity of *mdx* gastrocnemius and tibialis anterior muscles (Figure 4A). Both microdystrophins also significantly improved the specific force (force per cross sectional area of muscle) production in *mdx* muscles (*P*<0.05; Figure 4B). The specific force was not restored to wild-type partly because the sub-optimal dose of rAAV6-microdystrophin did not prevent the pseudo hypertrophy normally found in *mdx* muscles (*P* = 0.454 when comparing the muscle mass between *mdx* and treated *mdx* muscles; one-way ANOVA). Each microdystrophin significantly protected the treated limb muscles from contraction-induced injury (*P*<0.001; Figure 4C and 4D). However, we found no significant difference between the peak force, specific force or protection from contraction-induced injury when comparing between the two microdystrophins with either hinge 2 or hinge 3.

### Polyproline in hinge 2 influences the pathology of skeletal muscle fibers

**Figure 5:**
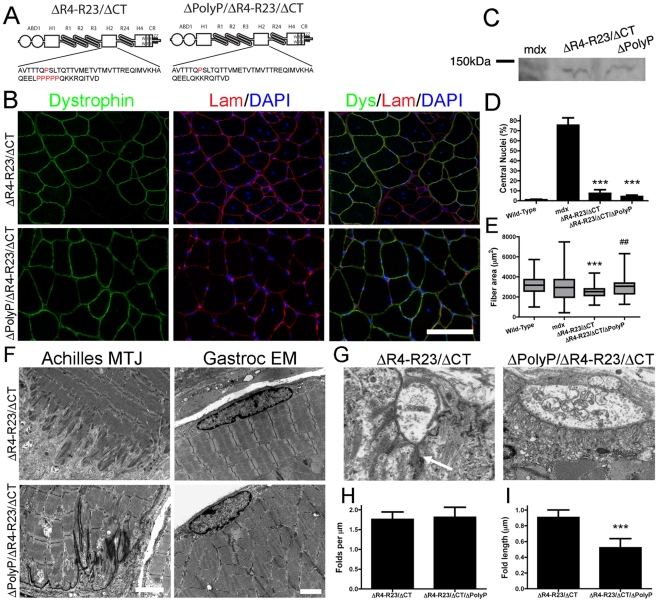
Microdystrophin^ΔR4-R23/ΔCT^ did not cause structural abnormalities when the polyproline site was deleted from hinge 2. (A) The molecular structure of microdystrophins. Below each structure is the amino acid sequence of hinge 2. Note that proline residues are highlighted in red and that microdystrophin^ΔPolyP/ΔR4-R23/ΔCT^ lacks the polyproline site. (B) Transverse sections of gastrocnemius muscles from *mdx* mice expressing microdystrophin^ΔR4-R23/ΔCT^ (top panel) or microdystrophin^ΔPolyP/ΔR4-R23/ΔCT^ (lower panel). Scale bar  = 100 µm. (C) Expression of truncated dystrophins from treated gastrocnemius muscles. The western blots were performed using tissue sections frozen in OCT. (D) Shown is the mean +/− S.D. percentage of myofibers with centrally-located nuclei. The treated muscles show the mean +/− SD for dystrophin positive fibers only. ***P<0.001 compared to untreated *mdx* mice. (E) The polyproline region influenced muscle fiber cross sectional area. Shown is the mean +/− distribution (25th and 75th percentile (box) in addition to the farthest (whiskers) of muscle fibers. ***P<0.001 compared to wild-type. ^##^ *P*<0.01 compared to microdystrophin^ΔR4-R23/ΔCT^. (F) Microdystrophin^ΔR4-R23/ΔCT^ expression led to a disruption of the myotendinous junctions and ringed fiber formation (arrow; top panels) in *mdx* gastrocnemius muscles, but not when the polyproline site was deleted (lower panels). Scale bars  = 2 µm. (G) Electron microscopy images of neuromuscular junctions (NMJ) in *mdx* muscles treated with rAAV6-microdystrophin^ΔR4-R23/ΔCT^ or rAAV6- microdystrophin^ΔPolyP/ΔR4-R23/ΔCT^. Scale bar  = 1 µm. (H) The graph shows the mean +/− SD number of folds per mm of postsynaptic membrane juxtaposed to the presynaptic cleft. (I) Deletion of the polyproline site from microdystrophin^ΔR4-R23/ΔCT^ restored the normal mean +/− SD length of synaptic folds (***P<0.001 compared to microdystrophin^ΔR4-R23/ΔCT^).

Together, our results suggested that the structural abnormalities observed in some treated *mdx* muscles could be traced to the presence of hinge 2 within the microdystrophin. We next examined the molecular composition of the hinges to define what was unique about hinge 2. The hinges in dystrophin are defined as such because of the higher concentration of proline residues, which function to limit the continuation of the α-helical coiled-coils of the spectrin-like repeats through the entire length of the dystrophin rod domain [19]. Both hinge 2 and hinge 3 have six proline residues and the lengths of these hinges are similar [19]. We hypothesized that the placement of the prolines most likely results in their different functions [5], [19]. Hinge 2 has 5 consecutive proline residues (polyproline; Figure 5A) whereas the proline residues in hinge 3 are more evenly distributed throughout the hinge [19]. Polyproline residues are thought to have their own defined rigid helical structure [41], [42], and this could affect the functional capacity of microdystrophin^ΔR4-R23/ΔCT^.

To test this hypothesis we compared muscles expressing the original microdystrophin^ΔR4-R23/ΔCT^ with a newly developed microdystrophin^ΔpolyP/ΔR4-R23/ΔCT^ that lacks the polyproline site in hinge 2 (Figure 5A). We delivered 6×10^10^ vg of each microdystrophin into *mdx* gastrocnemius muscles at 2 days of age and examined the mice 7 weeks after treatment. Both microdystrophins were expressed in a similar percentage of muscle fibers (Figure 5B; 59–68%), and were expressed at similar levels (Figure 5C). Each microdystrophin significantly reduced muscle fiber degeneration (Figure 5D). As expected, the original microdystrophin^ΔR4-R23/ΔCT^ limited muscle fiber cross-sectional area (Figure 5E), was associated with disrupted myotendinous junctions (Figure 5F), led to the formation of ringed fibers (Figure 5F), and perturbed neuromuscular junctions (Figure 5G–5I). In contrast, the *mdx* muscles treated with microdystrophin^ΔpolyP/ΔR4-R23/ΔCT^ did not show any abnormalities in muscle fiber maturation or structure (Figure 5). Thus, the presence of this polyproline site in hinge 2 of microdystrophin^ΔR4-R23/ΔCT^ prevented the appropriate integration of muscles into the nerve-tendon environment.

## Discussion

Most gene therapy strategies for DMD require the generation of highly functional truncated dystrophins. rAAV is an efficient and safe vector for systemically delivering truncated dystrophins to striated muscles to prevent muscle degeneration in animal models of DMD ([28]; reviewed in [43]). We had previously generated a microdystrophin^ΔR4-R23^ that was highly capable of mitigating muscle degeneration and improving the mechanical function of *mdx* skeletal muscles [24], [28]. However, the microdystrophin^ΔR4-R23^ transgene leads to chronic strain injury at the Achilles myotendinous junction [33]. This led to the formation of ringed fibers that function to protect skeletal muscles from contraction-induced injury, even better than wild-type mice [33]. The formation of the rings led to fragmentation of the neuromuscular junctions [36]. Other effects of the transgene included smaller muscle fibers [24], and increased length of synaptic folds [36]. Here we found that each of these phenotypic changes was recapitulated in *mdx* gastrocnemius muscles treated with rAAV6-microdystrophin^ΔR4-R23/ΔCT^. A screen of several newly developed dystrophin mini-genes revealed that the hinge 2 region influenced the functional capacity of microdystrophin^ΔR4-R23/ΔCT^. Replacing hinge 2 with hinge 3 led to several advantages such as better protection of skeletal muscles (only 1–2% central nuclei 6 months post treatment), larger muscle fibers and normal junctions. Deleting the polyproline site from hinge 2 of microdystrophin^ΔR4-R23/ΔCT^ also prevented these structural abnormalities.

### Mechanical properties of skeletal muscles expressing microdystrophins

Microdystrophin^ΔH2-R23+H3/ΔCT^ with hinge 3 significantly increased peak force, specific force and protected muscles from contraction-induced injury. However, the morphological improvements of microdystrophin^ΔH2-R23+H3/ΔCT^ treated muscles did not translate into a functional improvement compared to microdystrophin^ΔR4-R23/ΔCT^ treated muscles. This could result from the molecular and cellular responses to myotendinous strain injury that help protect the rAAV6-microdystrophin^ΔR4-R23/ΔCT^ treated muscles from contraction-induced injury [33]. Another possibility is that the presence of some dystrophin negative fibers masked any functional difference between the two proteins. The inclusion of hinge 2 in microdystrophin limited muscle fiber area whereas the inclusion of hinge 3 increased muscle fiber area (Figure 1). Larger muscle fibers in microdystrophin^ΔH2-R23+H3/ΔCT^ treated mice could have two distinct advantages: They could replace some of the muscle mass lost in advanced stages of disease and they could be better protected from contraction-induced injury [44]. However, the sub-optimal dose of either rAAV6-microdystrophin did not prevent the pseudo hypertrophy in *mdx* mice and no mechanical advantages could be discerned when comparing treatments. Saturating levels of rAAV6-microdystrophins or transgenic mice will most likely be required to detect minor differences in the mechanical properties of muscles expressing various truncated dystrophins.

### How does polyproline influence the functional capacity of truncated dystrophins?

Our most effective truncated dystrophins developed for gene therapy have been designed to maximize functional interactions between specific spectrin-like repeats and hinge domains. This design has been influenced by genetic studies in mice and man as well as biophysical studies *in vitro* on the structure, folding and physical properties of both individual and tandemly expressed spectrin-like repeats and hinge domains [24], [45]–[52]. Individual spectrin-like repeats are not all interchangeable, and ones adjacent to hinges have distinct properties from those flanked by other spectrin-like repeats [21], [24], [47], [51], [52]. Also, spectrin-like repeats rarely function as isolated units [15], [24], [50]–[53]. Instead, they appear to fold into nested domains interrupted by various insertions (hinges) that disrupt the uniformity and rigidity of the spectrin-like repeat rod domain [24], [45]–[48], [53]–[55]. These interruptions appear important for the elastic and flexible structure that dystrophin requires in its role as a force transducer and shock absorber in muscle [56]–[59]. Our studies suggest that the most functional truncations of dystrophin retain a central hinge domain that is flanked by spectrin-like repeats found adjacent to a hinge in the wild-type dystrophin [24]. Disruption of this linkage could influence protein folding, stability and function leading to the variable phenotypes in patients associated with deletions at or near hinge 3, which is encoded on exons 50–51 [21].

**Figure 6:**
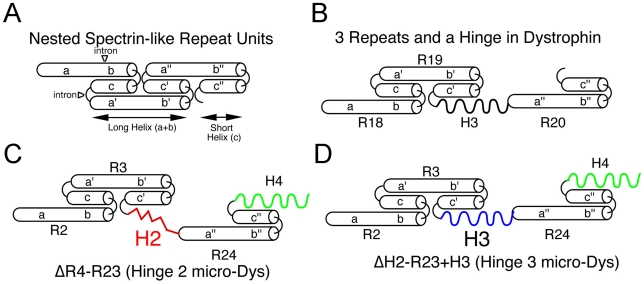
Predicted nested structure of specific dystrophin spectrin-like repeats relevant to microdystrophins and how they interact with hinge domains. (A) Predicted structure of 3 nested repeats [15]. Each repeat is composed of 3 helical domains (a, b, c) connected by non-helical linkers. The triple helical coiled-coil repeat structure is formed by helices b and c of the preceding repeat interfacing with the N-terminal helix a' from the following repeat. The helices a and b fold together into a long repeat, while helix c folds into the short repeat. (B) In dystrophin, the repeat domains are interrupted by hinge 2 (between repeats 3 and 4) and by hinge 3 (Repeats19 and 20). Shown is the predicted structure of the repeat 19-hinge 3-repeat 20 domain in full-length dystrophin. (C) The hinge 2-microdystrophin induces an unusual structural alteration that disrupts the normal connection between adjacent repeats. (D) In contrast, use of hinge 3 generates a similar structure as in (B) except that hinge 3 joins to repeats 3 and 24 rather than repeats 19 and 20.

Individual spectrin-like repeats are composed of 3 helical domains connected by non-helical linkers, which fold into a triple helical coiled coil structure (Figure 5A; [45], [47]). The linker regions between discreet repeats are also typically short and relatively unstructured to allow a smooth connection between the third helix of a preceding repeat and the first helix of the next repeat (Figure 6A). However, hinge domains interrupt the nested nature of adjacent spectrin-like repeats and allow more flexibility in the rod domain (Figure 6B). This degree of flexibility appears to be significantly different when hinge 2 or hinge 3 is present. While both hinges contain 6 prolines, which act to disrupt alpha helical structures, in hinge 3 they are dispersed whereas 5 of the 6 prolines in hinge 2 are clustered together (Figure 5A, Figure 6C and 6D; [10]). Polyproline residues form a rigid α-helix [41], [42], much like a molecular ruler [60]. We suggest that the location of this polyproline sequence within a highly truncated rod domain induces a severe structural disruption that can affect the ability of dystrophin to form a mechanically flexible connection between F-actin and β-dystroglycan. Spectrin-like repeats 1-3 have been shown to associate with the sarcolemmal membrane, while the WW domain in hinge 4 forms a critical portion of the β-dystroglycan binding domain [45], [61]. A rigid rod domain induced by polyproline in hinge 2 may directly impair the ability of microdystrophin to form a flexible interaction with either or both of these structures (Figure 6C). In contrast, when hinge 2 is present in full-length dystrophin, a significantly greater number of spectrin-like repeats are present between the hinge and the β-dystroglycan binding domain, allowing greater flexibility in the overall structure.

It is difficult to predict the function of the polyproline site from patients with in frame deletions of exon 17 (hinge 2) of dystrophin. The described deletions (Leiden Muscular Dystrophy Pages) usually encompass larger regions of dystrophin than the polyproline site and it is not clear how these deletions affect protein stability. Our finding that hinge 3 microdystrophin can prevent muscle degeneration suggests that the polyproline site is not a necessary component of dystrophin, similar to previous reports on longer forms of truncated dystrophins [24], [62].

Flanigan *et. al.,* 2009 has proposed that approximately 62% of all DMD patients could be treated with oligonucleotides that skip exons 45–55 (from spectrin-like repeat 18–22)[14]. This would create a truncated dystrophin that contains hinge 2 but not hinge 3, similar to, but much larger than our microdystrophin^ΔR4-R23^ transgene. It will therefore be of interest to determine whether the polyproline site in hinge 2 can influence the functional capacity of larger, truncated dystrophins. It will also be of interest to examine whether the polyproline site affects the functional capacity of truncated utrophin constructs that are designed for gene therapy of DMD [63], [64].

## Materials and Methods

### Mice and ethics statement

We utilized C57Bl/10 wild-type mice and *mdx^4cv^* mice. All experiments are in accordance with the institution of animal care and use committee (IACUC) of the University Of Washington.

### Generation of constructs

The expression vector CMV-ΔR4-R23/ΔCT which uses the cytomegalovirus immediate early promoter and enhancer to drive expression of a microdystrophin cDNA was generated as previously described [32]. We generated the ΔH2-R24/ΔCT, ΔR2-R23+R18-H3/ΔCT, ΔH2-R23+H3/ΔCT and ΔPolyP/ΔR4-R23/ΔCT constructs using recombination PCR with CMV-ΔR4-R23/ΔCT as the template [65]. The primers used to generate ΔH2-R24/ΔCT, ΔR2-R23+R18-H3/ΔCT, ΔH2-R23+H3/ΔCT and ΔPolyP/ΔR4-R23/ΔCT are found in Table S1. The resulting expression vectors were sequenced and co-transfected with the pDGM6 packaging plasmid into HEK293 cells to generate recombinant AAV vectors comprising serotype 6 capsids that were harvested, purified, and quantitated as described previously [29]. The resulting titer was determined by comparison to previously known concentrations of rAAV6-CMV-*lac*Z and ΔR4-R23/ΔCT by Southern analyses with a probe to the CMV promoter. The rAAV6-microdystrophins were delivered intravenously by tail vein injection at two weeks of age or directly into the *mdx* gastrocnemius muscles at 2 days of age while the mice were anaesthetized.

### Gross muscle morphology and morphometry

Gross muscle morphology was analyzed as previously described [24], [32]. Primary antibodies included the N-terminus of dystrophin (1∶800; [23]), utrophin A (1∶300; gift from Stanley Froehner, University of Washington), mouse monoclonal anti-α-dystrobrevin (Transduction laboratories; 1∶200), rabbit polyclonal anti-Syn17 (α-syntrophin; 1∶200; [66]), rabbit polyclonal anti-nNOS (Alexis; 1∶200). Secondary antibodies included Alexa 488, Alexa 594 rabbit polyclonal or Alexa 488 mouse monoclonal secondary antibodies (Molecular Probes; 1∶800). The sections were mounted in anti-fade mounting media containing DAPI (Vector Labs). Fluorescent sections were imaged using a Nikon eclipse E1000 fluorescent microscope (Nikon; NY) and captured using a DeltaVision fluorescence microscope. Muscle fiber areas were quantified using Image J (NIH).

### Immunoblotting

For immunoblots, n = 4 gastrocnemius muscles from *mdx* mice and *mdx* mice treated with rAAV6-microdystrophin^ΔR4-R23/ΔCT^ or rAAV6-microdystrophin^ΔH2-R23+H3/ΔCT^ were thawed from OCT blocks and placed into extract buffer (50 mM Tris-HCl, 150 mM NaCl, 0.2% sodium dodecyl sulfate, 10% glycerol, 24 mM Na Deoxycholate, 1% NP40, 47.6 mM Na Fluoride, 200 mM Na orthovanadate, Roche). Protein concentrations were determined by Coomassie Plus Bradford Assay (Peirce). Equal amounts of protein (15 mg) were resolved on a 4–12% SDS polyacrylamide gel. The blots were incubated in rabbit polyclonal antibodies to dystrophin (1∶500; kind gift from James Ervasti, University of Minnesota) and mouse monoclonal antibodies to α-sarcomeric actin (1∶500; SIGMA).

We also performed immunoblots on frozen tissue sections from n = 4 gastrocnemius muscles treated with rAAV6-microdystrophin^ΔR4-R23/ΔCT^ and microdystrophin^ΔPolyP/ΔR4-R23/ΔCT^ as previously described [67], with minor modifications. Briefly, we cut twenty-five 20 µm sections and diluted the sections into 200 µl lysis buffer (4% SDS, 25 mM Tris pH 8.8, 40% glycerol, 0.5 M phenylmethylsulfonyl fluoride, 100 mM dithiothreitol and bromophenol blue). Samples were briefly sonicated (10 sec at 4°C), heated to 95°C for 5 minutes, centrifuged for 5 minutes at 13,200×g and electrophoresed on a 4–12% SDS-polyacrylamide gel. The blots were incubated in primary rabbit polyclonal antibody against the N-terminus of dystrophin (1∶500; kind gift from James Ervasti, University of Minnesota). All blots were developed with ECL Plus (Pierce) and scanned with the Storm 860 imaging system (Amersham Biosciences).

### Electron microscopy

Electron microscopy was performed as previously described [33]. The junctional fold number and lengths were measured from n = 4 mice at 6 months of age using Image J (NIH) and compared using Students t-test (Prism). The counts represent the fold numbers and lengths from all fibers (dystrophin positive and negative).

### Quantitation of ringed fibers

We quantitated the number of ringed myofibers in EM images and thick (1 µm) toluidine blue sections from at least 4 animals per group. At least 300 muscle fibers from n = 4 gastrocnemius muscles were examined from wild-type, *mdx^4cv^* and *mdx^4cv^* mice expressing the various microdystrophins.

### Quantification of neuromuscular synapses

Neuromuscular synapses were analyzed in whole mount immunofluorescence stained muscles and quantitated as previously described [36]. The acetylcholine receptor clusters were stained with TRITC conjugated α-bungarotoxin (αBTX; 1∶800; Molecular Probes). Synapses were classified as continuous if they presented with 3 or less continuous regions of AChR clustering and discontinuous if they presented with more than 3 regions of AChR clustering. More than 50 synapses were analyzed from treated and untreated gastrocnemius skeletal muscle fibers from n = 4 mice. The counts in treated muscles include both dystrophin positive and negative fibers. We compared the proportion of continuous synapses using a Students t-test.

### Muscle physiology

Muscle physiology was performed as previously described for tibialis anterior [29] and gastrocnemius [33] muscles. We examined six-month-old wild-type, *mdx*, and *mdx* mice treated with rAAV6-microdystrophin^ΔR4-R23/ΔCT^ or rAAV6-microdystrophin^ΔH2-R23+H3/ΔCT^ (n = 5).

## Supporting Information

Figure S1 The molecular structure of truncated dystrophins. ABD1 at the N-terminus is composed of two calponin homology domains denoted by the two circles. The central rod domain contains 24 spectrin-like repeats (R1-24), 4 hinge domains, a 20 amino acid insertion between spectrin repeats 15 and 16, and a central actin-binding domain (ABD2). A cluster of basic repeats forms ABD2 that bind to actin through an electrostatic interaction. The hinge domains vary in that hinge 2 contains a polyproline site and hinge 4 contains a WW motif that is required for binding to dystroglycan. The cysteine rich region contains two EF hands and a ZZ domain that is also required for binding to dystroglycan. The microdystrophins used in this study are shown below the full-length dystrophin. Microdystrophin^ΔR4-R23/ΔCT^ has a large portion of the central rod domain missing between spectrin repeats 4 and 23 and also lacks the C-terminal domain (ΔR4-R23/ΔCT). Note that microdystrophin^ΔR4-R23/ΔCT^ and microdystrophin^ΔH2-R23+H3/ΔCT^ differ by a single hinge domain.
(9.25 MB TIF)

Figure S2 Expression of various microdystrophins in the gastrocnemius muscles of *mdx* mice significantly reduced the percentage of central nuclei. (A) Mean +/− S.D. of the percentage of muscle fibers expressing the microdystrophins. (B) Mean +/− S.D. percentage of central nuclei. ***P<0.001 compared to *mdx* mice.
(3.06 MB TIF)

Figure S3 Microdystrophin^ΔR4-R23/ΔCT^ leads to ringed fibers when hinge 2 is present. Shown are transverse sections from wild-type, *mdx* and *mdx* gastrocnemius muscles expressing various microdystrophins. Arrows point to ringed fibers in *mdx* muscles treated with microdystrophin^ΔR4-R23/ΔCT^. Scale bars  = 2 µm for electron microscopy images and 50 µm for the other images.
(9.93 MB TIF)

Figure S4 The hinge domains of dystrophin do not influence restoration of the dystrophin glycoprotein complex. Shown is the localization of dystrophin-associated proteins in transverse sections from wild-type, *mdx* and *mdx* mice treated with rAAV6-microdystrophin^ΔR4-R23/ΔCT^ or rAAV6-microdystrophin^ΔH2-R23/ΔCT+H3^. Scale bar  = 200 µm.
(10.31 MB TIF)

Table S1 Primers for cloning truncated dystrophin vectors
(0.04 MB DOC)

Text S1 Supporting details for supplemental data.
(3.54 MB DOC)
