# Supplementary figures and images for: The Polyproline Site in Hinge 2 Influences the Functional Capacity of Truncated Dystrophins
Source: PLoS Genet. 2010 May 20;6(5):e1000958. doi: 10.1371/journal.pgen.1000958 (PMC2873924; doi:10.1371/journal.pgen.1000958)

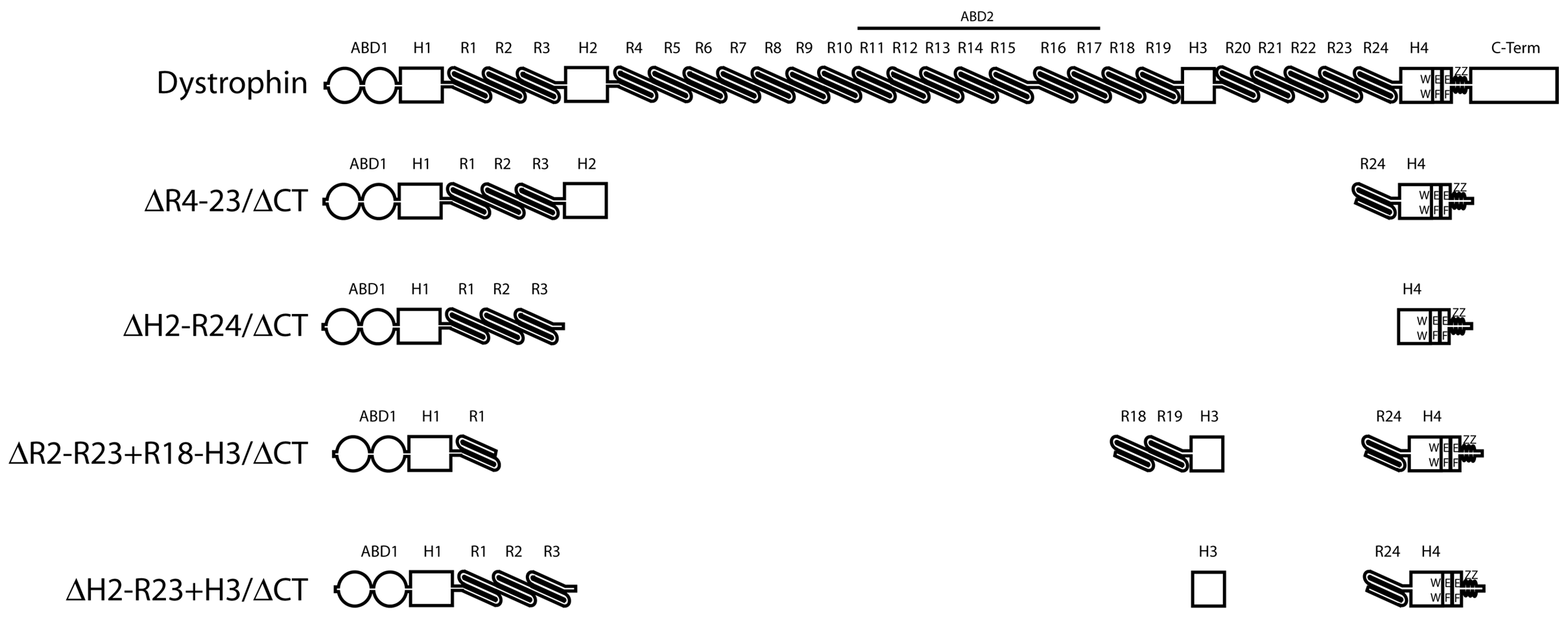

Supplement: Figure S1 — The molecular structure of truncated dystrophins. ABD1 at the N-terminus is composed of two calponin homology domains denoted by the two circles. The central rod domain contains 24 spectrin-like repeats (R1-24), 4 hinge domains, a 20 amino acid insertion between spectrin repeats 15 and 16, and a central actin-binding domain (ABD2). A cluster of basic repeats forms ABD2 that bind to actin through an electrostatic interaction. The hinge domains vary in that hinge 2 contains a polyproline site and hinge 4 contains a WW motif that is required for binding to dystroglycan. The cysteine rich region contains two EF hands and a ZZ domain that is also required for binding to dystroglycan. The microdystrophins used in this study are shown below the full-length dystrophin. MicrodystrophinΔR4-R23/ΔCT has a large portion of the central rod domain missing between spectrin repeats 4 and 23 and also lacks the C-terminal domain (ΔR4-R23/ΔCT). Note that microdystrophinΔR4-R23/ΔCT and microdystrophinΔH2-R23+H3/ΔCT differ by a single hinge domain. (9.25 MB TIF) [file pgen.1000958.s001.tif]

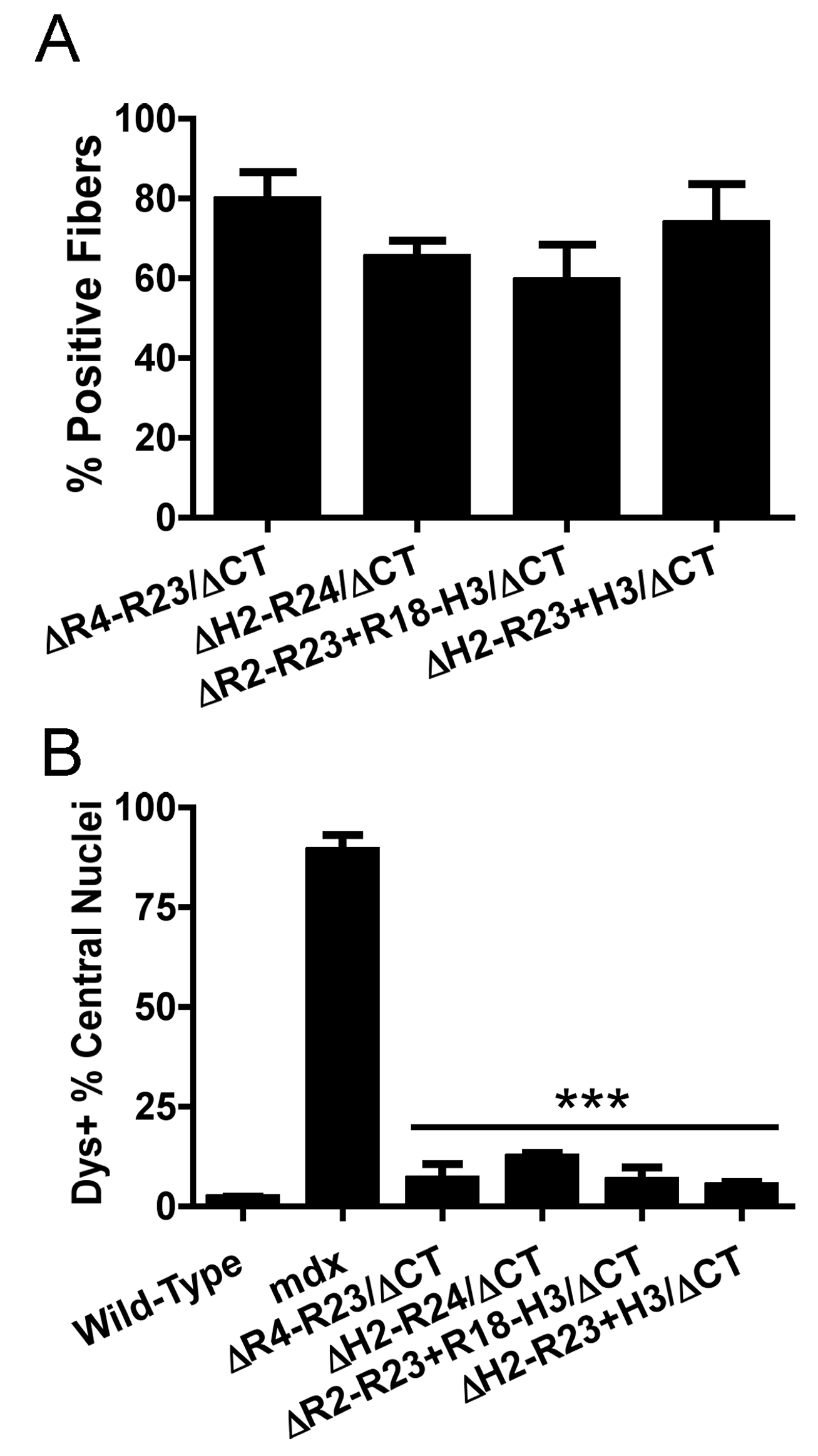

Supplement: Figure S2 — Expression of various microdystrophins in the gastrocnemius muscles of mdx mice significantly reduced the percentage of central nuclei. (A) Mean +/− S.D. of the percentage of muscle fibers expressing the microdystrophins. (B) Mean +/− S.D. percentage of central nuclei. ***P<0.001 compared to mdx mice. (3.06 MB TIF) [file pgen.1000958.s002.tif]

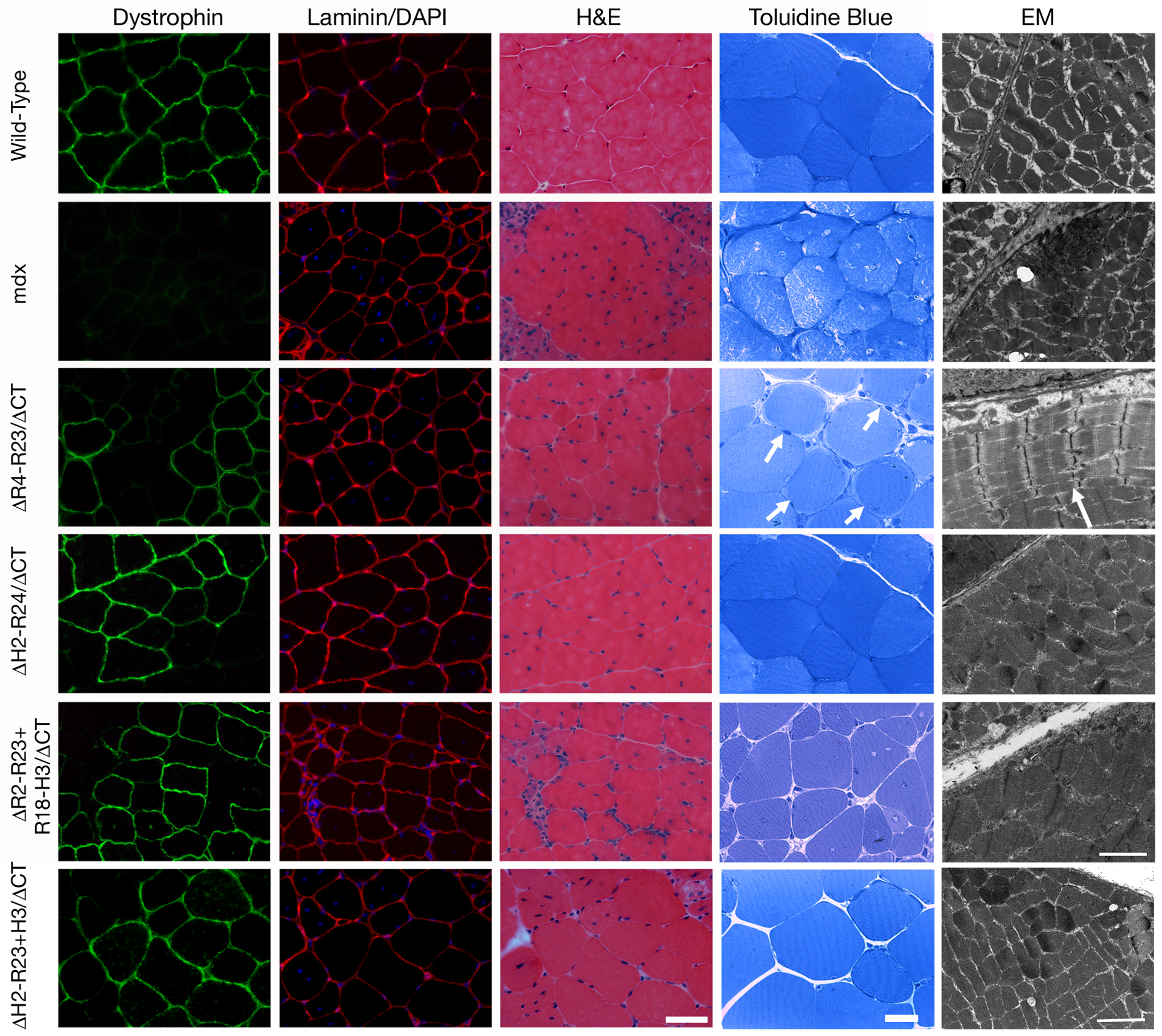

Supplement: Figure S3 — MicrodystrophinΔR4-R23/ΔCT leads to ringed fibers when hinge 2 is present. Shown are transverse sections from wild-type, mdx and mdx gastrocnemius muscles expressing various microdystrophins. Arrows point to ringed fibers in mdx muscles treated with microdystrophinΔR4-R23/ΔCT. Scale bars = 2 µm for electron microscopy images and 50 µm for the other images. (9.93 MB TIF) [file pgen.1000958.s003.tif]

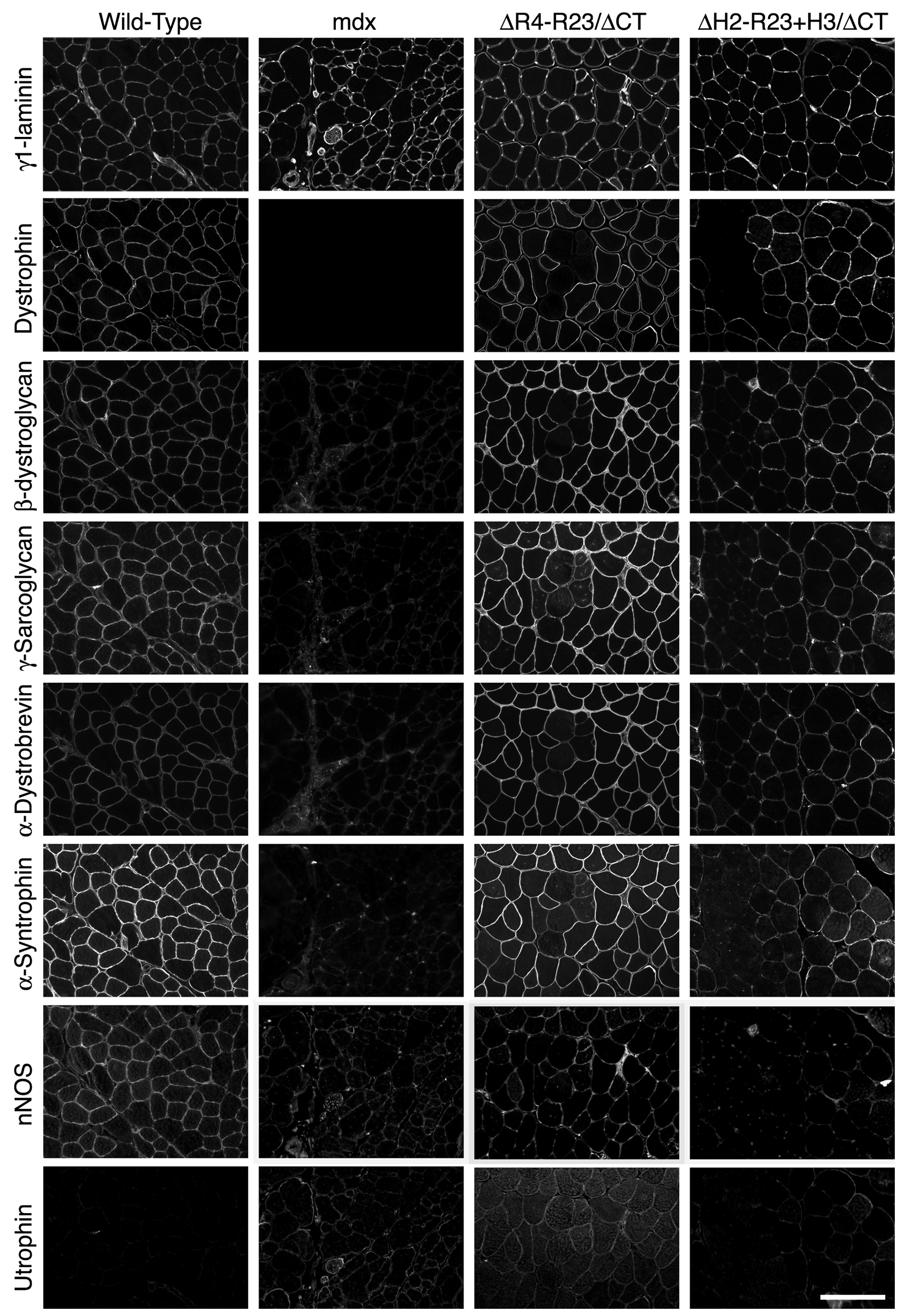

Supplement: Figure S4 — The hinge domains of dystrophin do not influence restoration of the dystrophin glycoprotein complex. Shown is the localization of dystrophin-associated proteins in transverse sections from wild-type, mdx and mdx mice treated with rAAV6-microdystrophinΔR4-R23/ΔCT or rAAV6-microdystrophinΔH2-R23/ΔCT+H3. Scale bar = 200 µm. (10.31 MB TIF) [file pgen.1000958.s004.tif]
