# Supplementary material for: The Polyproline Site in Hinge 2 Influences the Functional Capacity of Truncated Dystrophins
Source: PLoS Genet. 2010 May 20;6(5):e1000958. doi: 10.1371/journal.pgen.1000958 (PMC2873924; doi:10.1371/journal.pgen.1000958)
Supplement: Table S1 — Primers for cloning truncated dystrophin vectors (0.04 MB DOC) [file pgen.1000958.s005.doc]

**Table S1.** Primers for cloning truncated dystrophin vectors

| Truncated Dystrophin | Primer Sequences |
| --- | --- |
| ΔH2-R24/ΔCT: | 1/5’-GAA CAA TGT CAA CAA GGC AC-3’  2/5’-GAT GCT GGA CCA AAG TCC CTG TGG GCA GCC TGT GAA ATC TGT GC-3’ |
| ΔR2-17,20-23/ΔCT:  ΔH2-R23+H3/ΔCT  ΔPolyP/ΔR4-R23/ΔCT | 1/5’-GGA ATG CCT CAG GGT AGC TAG CAT GGA AAA ACA AAG CAA TTT ACA TAA GAA TAT CTT GTC AGA ATT TC-3’  2/5’-GAG TCT TTC AAG CTC CAA CAT CAA GGA AGA TGG-3’  3/5’-GAT GTT GGA GCT TGA AAG ACT CCA GGA ACT TC-3’  4/5’-TGT TTG GCG AGA TGG CTC-3’  1/5’-CTGCTGCTTTGGAAGAAC-3’  2/5’-ATAGTGGTCAGTCCAGGAGCAGCCTGTGAAATCTGTGCTGTAC-3’  3/5’-GCCATCTTCCTTGATGTTGGAGCTTGAAAGACTCCAGGAACTTC-3’  4/5’-GTGCCTTGACTTTCTCGAGGTGAT-3’  5/5’-GTACAGCACAGATTTCACAGGCTGCTCCTGGACTGACCACTATTGG-3’  6/5’-GTTGTTCCTGGAGTCTTTCAAGACTCCAACATCAAGGAAGATGGC-3’  1/5’-CTGCTGCTTTGGAAGAAC-3’  2/5’-CTGCCTCTTCTTTTGAAGTTCCTCTTGAGCATGCTTTA-3’  3/5’-ATGCTCAAGAGGAACTTCAAAAGAAGAGGCAGATTACTGT-3’  4/5’-CTGCAGAAGCTTCCATCTGGT-3’ |
