# Supplementary material for: The Polyproline Site in Hinge 2 Influences the Functional Capacity of Truncated Dystrophins
Source: PLoS Genet. 2010 May 20;6(5):e1000958. doi: 10.1371/journal.pgen.1000958 (PMC2873924; doi:10.1371/journal.pgen.1000958)
Supplement: Text S1 — Supporting details for supplemental data. (3.54 MB DOC) [file pgen.1000958.s006.doc]

**Supplement**

*Microdystrophin expression led to formation of ringed fibers when hinge 2 was present*

We began by examining ringed fibers, because they are the most straightforward way to detect major structural abnormalities in skeletal muscles across a reasonably large number of fibers. The first microdystrophin we generated (ΔH2-R24/ΔCT) was smaller than the original microdystrophin^ΔR4-R23/ΔCT^ in that spectrin-like repeat 24 and hinge 2 were deleted (Fig. S1). The second microdystrophin (ΔR2-R23+R18-H3/ΔCT) was similar in size to the original microdystrophin^ΔR4-R23/ΔCT^ but contained two different spectrin-like repeats and included hinge 3 rather than hinge 2 (Fig. S1). The third microdystrophin (ΔH2-R23+H3/ΔCT) was the same as the original microdystrophin^ΔR4-R23/ΔCT^ except that hinge 2 was replaced by hinge 3 (Fig. S1). Each newly developed microdystrophin lacked the C-terminal domain to accommodate the limited cloning capacity of rAAV (Fig. S1).

We administered 6x10^10^ vector genomes of an rAAV6 pseudotyped vector expressing each microdystrophin into the gastrocnemius muscles of 2 week-old *mdx* mice as previously described (1). Eleven weeks after injection, the microdystrophins were expressed in 60-80% of the *mdx* muscle fibers and each microdystrophin significantly reduced the percentage of myofibers with centrally-located nuclei (*P* < 0.001; Fig. S2). We next looked for ringed fibers using transverse resin sections stained with toluidine blue and electron microscopy (Fig. S3). Only the gastrocnemius muscles administered with rAAV6-microdystrophin^ΔR4-R23/ΔCT^ contained ringed fibers (Fig. S3). We found rings in approximately 8% of the microdystrophin^ΔR4-R23/ΔCT^ treated muscles fibers. We found no ringed fibers in wild-type, *mdx* or *mdx* muscles treated with any of the other microdystrophins including the microdystrophin^ΔH2-R23+H3/ΔCT^ which was only modified by switching hinge 2 with hinge 3 (Fig. S3). Thus, inclusion of hinge 2 in microdystrophin^ΔR4-R23/ΔCT^ led to rings in a subset of *mdx* gastrocnemius muscle fibers.

*The hinge regions do not influence restoration of the dystrophin-associated proteins*

Dystrophin provides a flexible connection between the cytoskeleton and the dystrophin-glycoprotein complex (2). The absence of dystrophin in *mdx* mice leads to a reduction of DGC components from the sarcolemma (Fig. S4) (3, 4). Microdystrophin^ΔR4-R23/ΔCT^ prevents the reduction of all DGC components from the *mdx* sarcolemma except for neuronal nitric oxide synthase (Fig. S4), as previously described (1, 5). Utrophin is normally found at the postsynaptic membrane in wild-type skeletal muscles (6), but is localized to the sarcolemma in *mdx* mice where it compensates for the lack of dystrophin (3, 4). Microdystrophin^ΔR4-R23/ΔCT^ does not prevent utrophin from localizing to the sarcolemma in treated *mdx* mice (Fig. S4). We found no difference in the localization of dystrophin-associated proteins components when comparing microdystrophin^ΔR4-R23/ΔCT^ to microdystrophin^ΔH2-R23+H3/ΔCT^ in the *mdx* gastrocnemius muscles. Thus, hinge 2 and hinge 3 of dystrophin had no influence on the dystrophin-associated protein composition at the sarcolemma in *mdx* muscles treated with microdystrophins.

**References**

1. Yue, Y., Liu, M. and Duan, D. (2006) C-terminal-truncated microdystrophin recruits dystrobrevin and syntrophin to the dystrophin-associated glycoprotein complex and reduces muscular dystrophy in symptomatic utrophin/dystrophin double-knockout mice. *Mol Ther,* 14, 79-87.

2. B Banks GB and Chamberlain JS (2008). The value of mammalian models for Duchenne muscular dystrophy in developing therapeutic strategies. Curr. Top. Dev. Biol. *In Press*.

3. Deconinck, A.E., Rafael, J.A., Skinner, J.A., Brown, S.C., Potter, A.C., Metzinger, L., Watt, D.J., Dickson, J.G., Tinsley, J.M. and Davies, K.E. (1997) Utrophin-dystrophin-deficient mice as a model for Duchenne muscular dystrophy. *Cell,* 90, 717-27.

4. Grady, R.M., Teng, H., Nichol, M.C., Cunningham, J.C., Wilkinson, R.S. and Sanes, J.R. (1997) Skeletal and cardiac myopathies in mice lacking utrophin and dystrophin: a model for Duchenne muscular dystrophy. *Cell,* 90, 729-38.

5. Harper, S.Q., Crawford, R.W., DelloRusso, C. and Chamberlain, J.S. (2002) Spectrin-like repeats from dystrophin and alpha-actinin-2 are not functionally interchangeable. *Hum Mol Genet,* 11, 1807-15.

6. Ohlendieck, K., Ervasti, J.M., Matsumura, K., Kahl, S.D., Leveille, C.J. and Campbell, K.P. (1991) Dystrophin-related protein is localized to neuromuscular junctions of adult skeletal muscle. *Neuron,* 7, 499-508.

7. Amann, K.J., Renley, B.A. and Ervasti, J.M. (1998) A cluster of basic repeats in the dystrophin rod domain binds F-actin through an electrostatic interaction. *J Biol Chem,* 273, 28419-23.

8. Ervasti, J.M., Ohlendieck, K., Kahl, S.D., Gaver, M.G. and Campbell, K.P. (1990) Deficiency of a glycoprotein component of the dystrophin complex in dystrophic muscle. *Nature,* 345, 315-9.

9. Koenig, M. and Kunkel, L.M. (1990) Detailed analysis of the repeat domain of dystrophin reveals four potential hinge segments that may confer flexibility. *J Biol Chem,* 265, 4560-6.

10. Harper, S.Q., Hauser, M.A., DelloRusso, C., Duan, D., Crawford, R.W., Phelps, S.F., Harper, H.A., Robinson, A.S., Engelhardt, J.F., Brooks, S.V. *et al.* (2002) Modular flexibility of dystrophin: implications for gene therapy of Duchenne muscular dystrophy. *Nat Med,* 8, 253-61.
